# Metabolite profiling and free radical scavenging activity studies of alkaloids from *Erythrina crista-galli* twigs through in vitro and in silico analysis

**DOI:** 10.1038/s41598-025-17130-x

**Published:** 2025-10-02

**Authors:** Abd. Wahid Rizaldi Akili, Nandang Permadi, Ari Hardianto, Astin Lukum, Jalifah Latip, Tati Herlina

**Affiliations:** 1 Department of Chemistry, Faculty of Mathematics and Natural Science, Universitas Padjadjaran, 45363 Jatinangor, Sumedang, West Java Indonesia; 2 Graduate School, Universitas Padjadjaran, 40132 Bandung, West Java Indonesia; 3 Department of Chemistry, Faculty of Mathematics and Natural Science, Universitas Negeri Gorontalo, Gorontalo, 96554 Indonesia; 4 Department of Chemical Sciences, Faculty of Science and Technology, Universiti Kebangsaan Malaysia (UKM), 46300 Bangi, Selangor Malaysia

**Keywords:** *Erythrina crista-galli*, Metabolite profiling, Erythrina alkaloids, Antioxidant, Density functional theory, Plant sciences, Secondary metabolism, Cheminformatics, Theoretical chemistry

## Abstract

*Erythrina crista-galli* is among 130 species of genus *Erythrina* which widely distributed in Indonesia, Australia, Argentina, Uruguay, Paraguay, and Brazil. Among the botanical parts used for the exploration of alkaloids from *E. crista-galli* (cockspur coral, ceibo, corticeira), the twig being one that rarely explored. This study aims to explore the alkaloid content from the twig of *E. crista-galli* as well as to evaluate their free radical scavenging activities through in vitro and in silico studies. The metabolite profile of the ethanol extract from *Erythrina crista-galli* twigs was characterized using Ultra-Performance Liquid Chromatography coupled with Tandem Mass Spectrometry (UPLC-MS/MS). The structures of the isolated alkaloids were determined through Nuclear Magnetic Resonance (NMR) and Mass Spectrometry (MS). Their free radical scavenging activities were evaluated using the 1,1-diphenyl-2-picrylhydrazyl (DPPH) free radical scavenging assay, while their electronic properties were assessed via Density Functional Theory (DFT). Twelve erythrina type alkaloids were tentatively identified from the twig of *E. crista-galli*. Four among these alkaloids, namely crystamidine (1), 8-oxoerythraline (2), erythrinine (3), erythraline (4) were isolated and their structures were confirmed through NMR and MS spectroscpy. Among the isolated alkaloids, erythraline (4) show highest free radical scavenging activity with IC_50_ of 182.5 ± 5.3 µg/mL, whereas 8-oxoerythraline (1) show the lowest free radical scavenging activity with IC_50_ of 868.2 ± 0.26 µg/mL. Structure–Activity relationship study shows that any modification in erythraline base skeleton, would decrease the antioxidant activity of erythraline, Furthermore, DFT calculation revealed that erythrinine (3), erythraline (4) tend to act as electron donors, whereas crystamidine (1), 8-oxoerythraline (2) tend to act as electron acceptors.

**Supplementary Information:**

The online version contains supplementary material available at 10.1038/s41598-025-17130-x.

## Introduction

Alkaloids are naturally occurring secondary metabolites characterized by the presence of nitrogen in their molecular structure. They are mainly found in plants and are abundant in certain families of flowering plants^1^. Alkaloids make up approximately 20% of the known secondary metabolites founds in plants, and are well known to play significant role in both human medicine and in an organism’s natural defence^2^. In human medicine, they are particularly well known as anaesthetics, cardioprotective, and anti-inflammatory agents^3^, whereas in plants, they play role to enhance reproductive rate by improving plants defence against predators^4^.

The plants species of genus *Erythrina* are flowering plants have widely recognised as natural sources of bioactive alkaloids with wide range of biological activity^5–7^. In addition to their alkaloids content with various structural diversity, plants of genus *Erythrina* are known as natural sources of antioxidant compounds^8^. Extracts from various *Erythrina* species, including *E. indica*, *E. variegata*, *E. senegalensis*, *E. stricta*, and *E. crista-galli* have reported to exhibit significant antioxidant activities^9–13^. Moreover, a number of alkaloids isolated from genus *Erythrina*, such as hypaphorine, erysodine, erythraline, erythrartine, erysotramidine, erythrinine, and cristanine A have shown promising potential as antioxidants^14–18^.

*Erythrina crista-galli* (cockspur coral, ceibo, corticeira) is one among 130 species of genus *Erythrina* which widely distributed in Indonesia, Australia, Argentina, Uruguay, Paraguay, and Brazil^11^. Among the botanical parts used for the exploration of alkaloids from *E. crista-galli*, the twig being one that rarely explored. To the best of our knowledge there is no available literature which focus on the exploration of alkaloids from the twig of *E. crista-galli*. Furthermore, while numerous studies have reported the potential antioxidant activity of alkaloids derived from *Erythrina*, there is still a lack of research investigating the electronic properties of these alkaloids to better understand their antioxidant activities. This study aims to bridge these gaps by exploring the alkaloid content of *E. crista-galli* twigs and evaluating their antioxidant properties through both in vitro and in silico approaches.

## Material and method

### Plant material

The twigs of *E. crista-galli* were collected from Sersan Badjuri St., Bandung, West Jawa, Indonesia. This plant materials have been determined at the Laboratory of Agricultural Production Technology & Services, Agricultural Cultivation Department, Faculty of Agriculture, University Padjadjaran voucher number 1020 and was identified by Joko Kusmoro. The collection and use of plant material in this study complied with all relevant institutional, national, and international guidelines and legislation.

### UPLC-MS/MS analysis

The chemical components in the extract from *E. crista-galli* twigs were separated using an Ultra Performance Liquid Chromatography (UPLC) system. The analysis was carried out with a C18 column (1.8 µm, 2.1 × 100 mm) at a column temperature of 50 °C and an ambient temperature of 25 °C. The mobile phase comprised water with 5 mM ammonium formate (phase A) and acetonitrile with 0.05% formic acid (phase B). A step gradient method was used over a total runtime of 23 min, with a flow rate of 0.2 mL/min. Prior to injection, samples were filtered through a 0.2 µm syringe filter, and an injection volume of 5 µL was utilized. Mass spectrometric analysis was conducted using an electrospray ionization (ESI) system. The mass analysis range covered 50 to 1200 m/z. The source temperature was kept at 100 °C, while the desolvation temperature was maintained at 350 °C with a gas flow rate of 793 L/hr. For low-energy analysis, the collision energy was set at 4 V, and a ramp collision energy between 25 and 60 V was utilized. Leucine enkephalin was infused every 10 s throughout the run for internal mass correction.

### Alkaloid extraction

The powdered twigs of *E. crista-galli* (9.8 kg) was submerged with ethanol for 3 × 24 h. The ethanol extract was filtered and evaporated in vacuo to give of concentrated ethanol extract. The concentrated ethanol (128,7 g) extract was dissolved in water and was acidified with acetic acid until the solution reach pH of 3. The acidic solution was then extracted with ethyl acetate three times. The acidic aqueous phase was then basified with NH_4_OH to reach the pH of 9, then were extracted again with ethyl acetate. The ethyl acetate phase was evaporated in vacuo to give crude alkaloidal extract (10.42 g).

### Alkaloid separation and isolation

The crude alkaloidal extract (10.42 g) from the twig of *E. crista-galli* were separated with coloumn chromatography using CHCl_3_ and EtOAc in a 10% gradient (10:0–5:5) to give eleven fractions (Fr.1–Fr.11). Fr. 6 (86.4 mg) were subjected to coloumn chromatography using n-Hexane: CHCl_3_: CH_3_OH (isocratic, 6:3;1) to give four subfraction (Fr. 6a–Fr. 6d). Subfraction 6a (24.7 mg) were subjected to coloum chromatography using CH_2_Cl_2_: Acetone in a 10% gradient (10:0 – 0:10) to give compound **1** (2.6 mg) and compound **2** (4.8 mg). Fr. 7 (1.119 g) were subjected to coloumn chromatography using CH_2_Cl_2_: EtOAc in a 5% gradient (10:0–6:4) to give four subfraction (Fr.7a–Fr.7d). Subfraction 7b (18.3 mg) were subjected to coloum chromatography using CH_2_Cl_2_: EtOAc in a 5% gradient (10:0–0:10) to give compound **3** (3.2 mg). Subfraction 7c (860 mg) were subjected to coloum chromatography using CH_2_Cl_2_: Acetone in a 10% gradient (10:0–0:10) to give compound **4** (3.9 mg) along with three subfraction (Fr. 7c2–Fr. 7c4).

### Antioxidant activity assay

The antioxidant activities of the isolated alkaloids were evaluated using DPPH free radical scavenging assay with 96 well microtiter plate as described in the literature^19^. The isolated compounds with various concentrations were prepared by dilution with methanol. Into a 96-well microplate, 200 μL of prepared 1.0 mM DPPH solution was mixed with 10 μL of the sample solution. The resulting solution was allowed to react for 30 min in the dark room at ambient temperature. After 30 min of reaction, the absorbance of the solution was measured at 517 nm using microplate reader. Ethanol was used as the blank solution, whereas ascorbic acid was used as positive control. The percentage of DPPH activity inhibition was calculated using the following equation

$$\text{\% Inhibition }= \frac{\left(Absorbance \,of \,blank\, solution\right)-(Absorbance\, of \,test sample)}{Absorbance\, of\, blank\, solution}{\times }100\text{\%}$$

The experiment was done in duplo, and the IC_50_ value was obtained from plotting the sample concentration in x axis against % inhibition in y axis.

### Density functional theory study

All calculations for the five isolated alkaloids were performed using the Gaussian 09 software (https://gaussian.com/). The most stable conformer of the isolated compounds was selected through potential energy scanning using DFT with the B3LYP method and 6-311G++ (d,p) basis set^20^. The structures of the five isolated alkaloids, including their ions and radicals, were optimized using the same basis set. All the optimized structures were accepted through vibrational frequency analysis (no imaginary frequency was found for the optimized structure). The global descriptive parameter, including ionisation potential (I), electron affinity (A), hardness (η), softness (S), electronegativity (χ), chemicalpotential (μ) and electrophilicity index (Ω) was calculated according to energy vertical method. The equation for each global descriptive parameter is given below^20^:

$${\text{I}} = {\text{E}}_{{{\text{cation}}}} {-}{\text{E}}_{{{\text{neutral}}}}$$

$${\text{A}} = {\text{E}}_{{{\text{neutral}}}} {-}{\text{E}}_{{{\text{anion}}}}$$

$$\upeta =\frac{(\text{I}-\text{A})}{2}$$

$$\upchi = \frac{(\text{I }+\text{ A})}{2}$$

$$\text{s}= \frac{1}{2\upeta }$$

$$\mu = - \, \chi$$

$$\upomega = \frac{{\upmu }^{2}}{2}$$

$$\upomega^{-} =\frac{({3\text{I}+\text{A})}^{2}}{16 (\text{I}-\text{A})}$$

$$\upomega^{+} = \frac{({\text{I}+3\text{A})}^{2}}{16 (\text{I}-\text{A})}$$

## Result and discussion

### Alkaloids profile of *E. crista-galli* twigs

*E. crista-galli* is one species of *Erythrina* plants known as biological source of alkaloids. While numerous studies have documented the isolation of *Erythrina* alkaloids from various parts of the *E. crista-galli* tree, including the flowers, stem bark, roots and leaves, there remains limited information regarding the alkaloid content specifically in the twigs. To address this research gap, UPLC-MS/MS analysis was conducted to uncover the metabolite profile, particularly alkaloids present in the twigs of *E. crista-galli*.

**Fig. 1:**
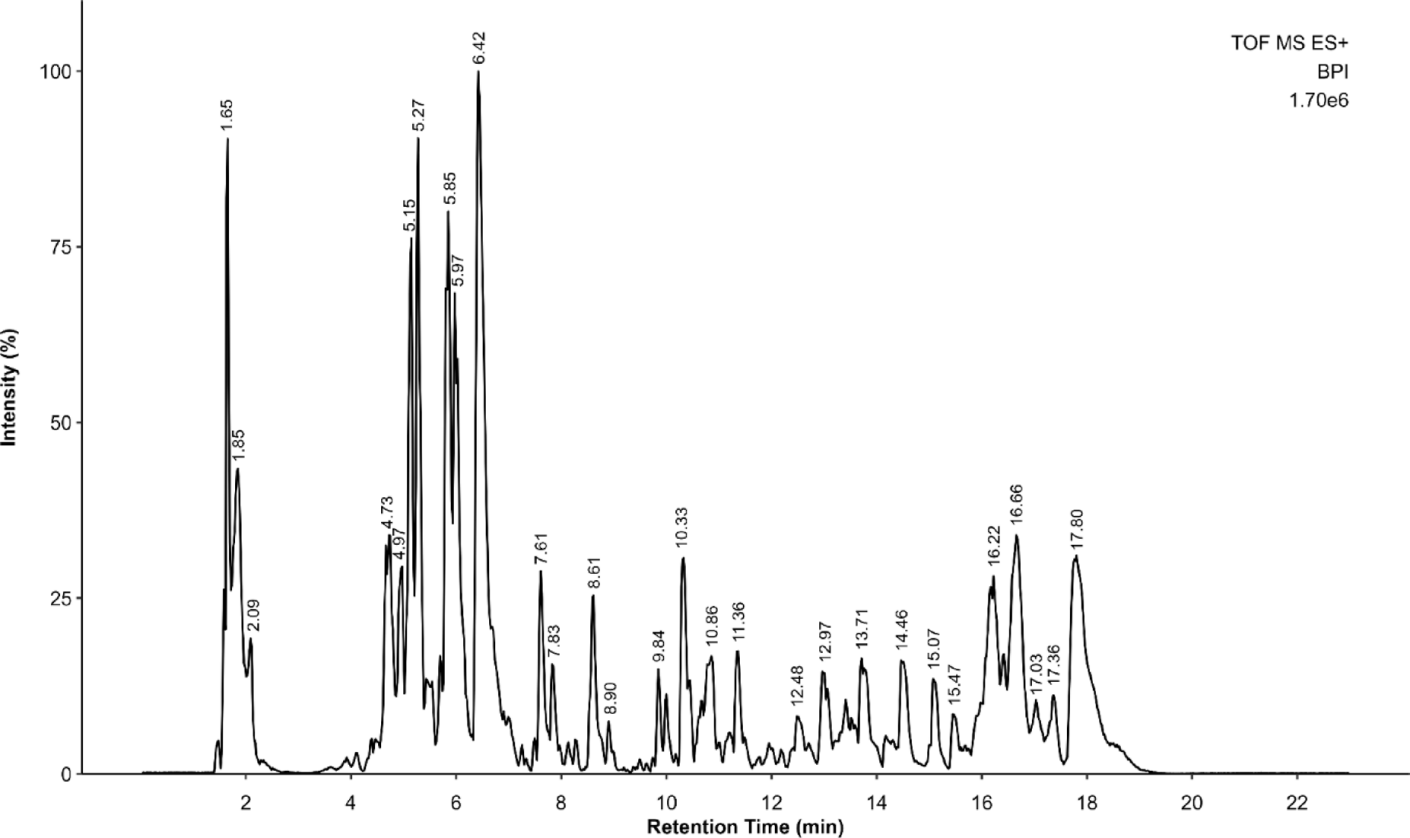
Chromatogram obtained from UPLC-MS/MS analysis in positive ion mode.

Interpretation of chromatogram and the mass spectrum from the positive ion mode (Fig. 1), has led to tentative identification of twelve erythrina alkaloids based on their observed *m/z* and comparison with available literatures online (Table 1). These erythrina alkaloids are comprised of four alkenoid-type erythrina alkaloids and eight dienoid-type erythrina alkaloids. Most of these alkaloids have been identified in *E. crista-galli*, except for 6-*O*-β-glucococcoline (RT 5.27), erythrinine N-oxide-11-O-β-D-glucose (RT 5.42), erythrinine N-oxide (RT 5.97), and 10,11‐Dioxoerythraline (RT 8.61).

**Table 1:** Erythrina alkaloids tentatively identified in ethanol extract of *E. crista-galli* twig.

tR (min)	m/z	Molecular formula	Mass error (ppm)	Tentative identification	Biological source in *Erythrina* Plants
4.66	332.1513	C_18_H_22_NO_5_^+^	4.5		*E. crista-galli*^16^
5.13	316.1556	C_18_H_22_NO_4_^+^	2.2		*E. crista galli*^16^
5.27	476.1917	C_24_H_30_NO_9_^+^	0.8		*E. velutina*^21^
5.42	492.1862	C_24_H_30_NO_10_^+^	1.6		*E. arborescens*^22^
5.69	346.1653	C_19_H_24_NO_5_^+^	0.3		*E. crista-galli*^23^
5.85	314.1393	C_18_H_20_NO_4_^+^	0.3		*E. crista-galli*^16^
5.97	330.1343	C_18_H_20_NO_5_^+^	0.6		*E. corallodendron*^24^
7.26	344.1503	C_19_H_22_NO_5_^+^	1.5		*E. crista galli*^25^
7.41	298.1439	C_18_H_20_NO_3_^+^	1.0		*E. crista galli*^16^
7.49	310.1087	C_18_H_16_NO_4_^+^	2.6		*E. crista-galli*^16^
8.61	326.1039	C_18_H_16_NO_5_^+^	3.4		*E. collarodendron*^26^
8.90	312.1243	C_18_H_18_NO_4_^+^	2.2		*E. crista-galli*^16^

### Alkaloids isolated from twig of *Erythrina crista-galli*

**Fig. 2:**
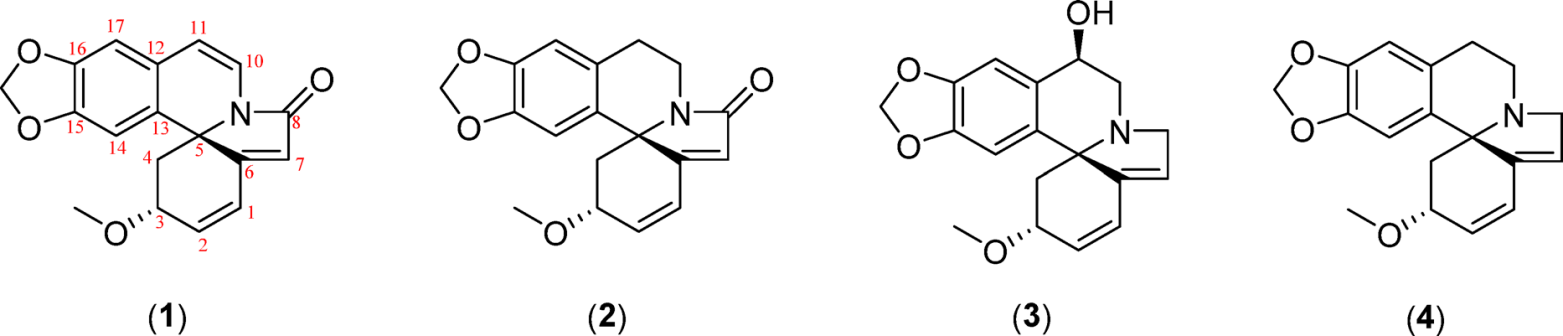
Chemical structures of compound 1–4.

Four erythrina alkaloids were successfully isolated from the twigs of *E. crista-galli*. The chemical structures of these alkaloids were elucidated using ^1^H and ^13^C NMR as well as a comparison of these data with available data from previous research. Based on their NMR spectra, compound **1** was identified as crystamidine^27^, compound **2** as 8-oxoerythraline^28^, compound **3** as erythrinine^29^, and compound **4** as erythraline^30^ (Fig. 2). The stereochemistry of C3 and C5 were assigned according to literature precedents, which consistently report that erythrina type alkaloids exhibit dextrorotatory optical activity and poses a 3R,5S stereochemistry^31^.

Compound **1** was obtained as yellow oil. The molecular formula was determined as C_18_H_16_NO_4_ by HR-TOF–MS (*m/z* 310.1072 [M + H]^+^, calculated for 310.1079). ^1^H NMR (400 MHz, acetone-d6) δH 1.33 (1H; dd; 11.32 Hz; 1.04 Hz); 2.68 (1H; m); 3.25 (3H; s); 3.71 (1H; m); 5.96 (1H; d; 1.04 Hz); 6.00 (1H; d; 1 Hz); 6.71 (1H; s); 6.86 (1H; s); 6.21 (1H; d; 7.24 Hz); 6.09 (1H; s); 6.83 (1H; d; 7.24 Hz); 7.05 (1H; dd; 10.2 Hz; 2.52 Hz); 6.42 (1H; dt). ^13^C NMR (500 MHz. acetone-d6) δC 42.7 (C1); 55.5 (C2); 65.7 (C3); 74.4 (C4); 101.5 (C5); 103.7 (C6); 107.4 (C7); 112.8 (C8); 119.7 (C9); 120.5 (C10); 123.5 (C11); 125.6 (C12); 126.4 (C13); 103.7 (C14); 146.7 (C15); 147.3 (C16); 107.4 (C17); 101.5 (-OCH_2_O-); 55.5 (3-OCH_3_).

Compound **2** was obtained as yellow oil. The molecular formula was determined as C_18_H_18_NO_4_ by HR-TOF–MS (*m/z* 312.1245 [M + H]^+^, calculated for 312.1236). ^1^H NMR (400 MHz. acetone-d6) δH 6.9 (1H. dd); 6.38 (1H; d; 10.2); 3.73 (1H. m); 1.57 (1H; dd; 10.2; 1.48); 2.90 (1H. m); 5.97 (1H; s); 3.55 (2H; m); 3.18 (1H; m); 2.96 (1H; m); 6.80 (1H; s); 6.73 (1H; s); 5.95 (1H; d; 1.04 Hz); 5.92 (1H; d; 1.04 Hz); 3.31 (3H; s). ^13^C NMR (500 MHz. acetone-d6) δC 124.0 (C1); 137.3 (C2); 75.4 (C3); 41.8 (C4); 66.9 (C5); 158.2 (C6); 120.3 (C7); 170.8 (C8); 38.0 (C10); 27.3 (C11); 128.4 (C12); 131.1 (C13); 105.2 (C14); 147.5 (C15); 146.5 (C16); 109.8 (C17); 101.7 (–OCH_2_O–); 56.1 (3-OCH_3_).

Compound **3** was obtained as brownish oil. The molecular formula was determined as C_18_H_20_NO_4_ by HR-TOF–MS (*m/z* 314.1396 [M + H]^+^, calculated for 314.1392). ^1^H NMR (400 MHz. acetone-d6) 6.54 (1H; d; 12 Hz); 5.97 (1H; d; 9.5 Hz); 3.93 (1H; brs); 1.78 (1H; d; 11 Hz); 2.35 (1H; d; 11.5 Hz); 5.72 (1H; s); 3.84 (2H; s); 2.97 (1H; dd; 13.25 Hz; 3.6 Hz); 3.56 (1H; dd; 14 Hz; 4.5 Hz); 4.69 (1H; t; 4.5 Hz); 6.77 (1H; s); 6.96 (1H; s); 5.91 (2H; s); 3.30 (3H; s); ^13^C NMR (500 MHz. acetone-d6) δC 125.4 (C1); 131.7 (C2); 76.0 (C3); 41.0 (C4); 67.0 (C5); 142.1 (C6); 123.5 (C7); 59.3 (C8); 51.7 (C10); 65.1 (C11); 130.2 (C12); 131.3 (C13); 106.0 (C14); 147.3 (C15); 146.8 (C16); 107.4 (C17); 101.1 (-OCH_2_O-); 56.2 (3-OCH_3_).

Compound **4** was obtained as yellow needle like crystal. The molecular formula was determined as C_18_H_20_NO_3_ by HR-TOF–MS (*m.z* 298.1439 [M + H]^+^, calculated for 298.1443). ^1^H NMR (400 MHz. acetone-d6) δH 6.53 (1H; dd; 9.75 Hz; 2 Hz); 5.92 (1H; dd; 5 Hz; 3.5 Hz); 3.94 (1H; m); 1.83 (1H; t; 11.5 Hz); 2.48 (1H; dd; 11.5 Hz; 6.0 Hz); 5.71 (1H; s); 3.49 (2H; m); 2.89 (2H. m). 2.5 (1H; dd; 11.5 Hz; 6 Hz); 2.7 (1H; dd; 10.5 Hz; 8.5); 6.75 (1H; s); 6.61 (1H; s); 5.89 (2H; d; 2 Hz); 3.31 (3H; s). ^13^C NMR (500 MHz. acetone-d6) δC 125.2 (C1); 131.7 (C2); 76.0 (C3); 41.2 (C4); 74.6 (C5); 142.1 (C6); 122.7 (C7); 57.6 (C8); 44.6 (C10); 25.2 (C11); 127.8 (C12); 131.7 (C13); 106.2 (C14); 146.2 (C15); 145.9 (C16); 108.7 (C17); 100.8 (-OCH_2_O-); 56.1 (3-OCH_3_).

### Free radical scavenging activities of isolated compounds

**Fig. 3:**
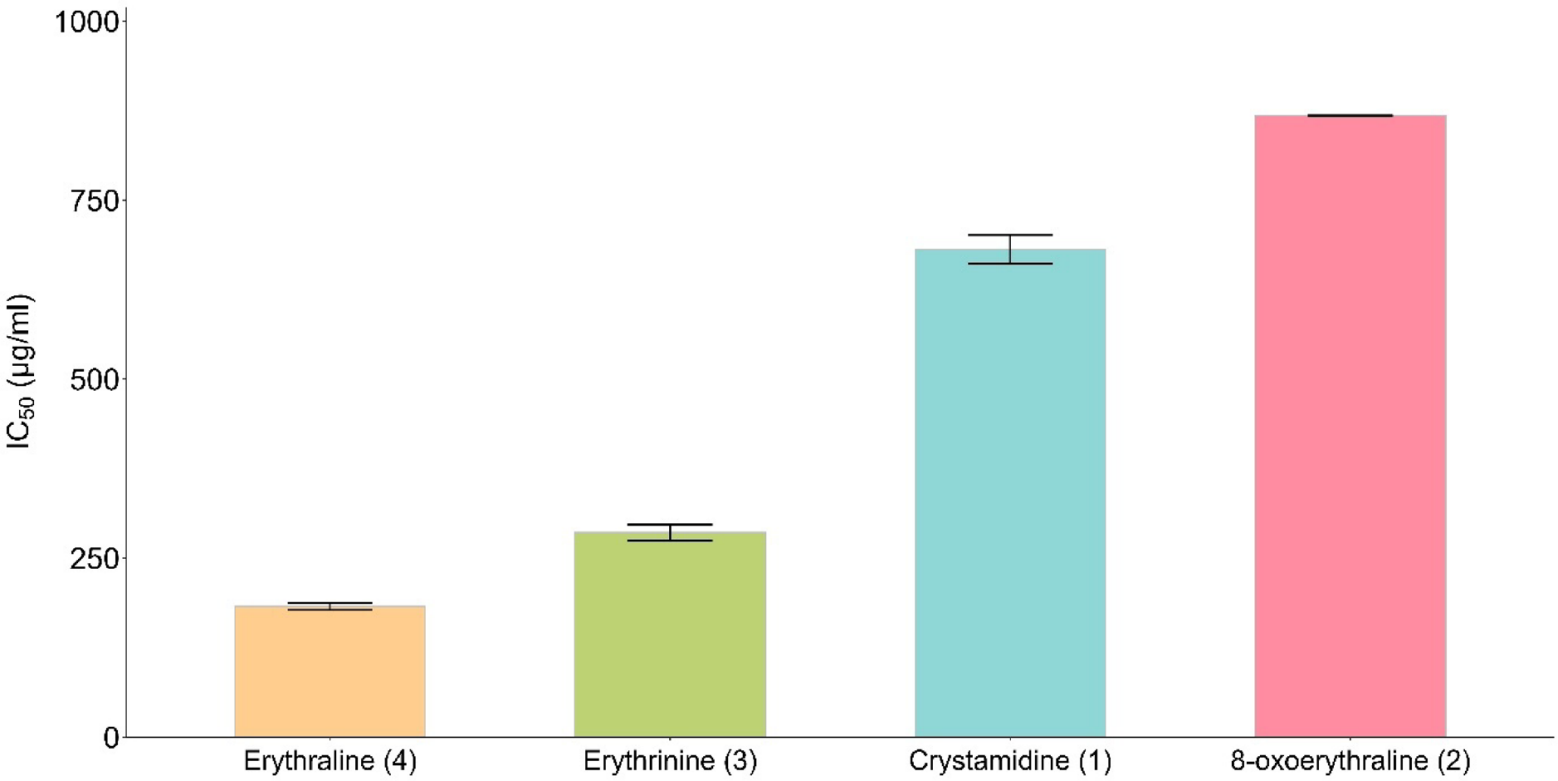
Free Radical Scavenging Activities of Compound **1**–**4**.

The free radical scavenging activities of the isolated compounds were evaluated using the 2,2-Diphenyl-1-picrylhydrazyl (DPPH) free radical scavenging test. Among the four erythrina alkaloids being evaluated, erythraline (4) exhibited the highest free radical scavenging activity with IC_50_ of 182.5 ± 5.3 µg/mL, this was followed by erythrinine (3), cristamidine (1), and 8-oxoerythraline (2) with IC_50_ of 285 ± 10.9 µg/mL, 681.4 ± 20.2 µg/mL, and 868.2 ± 0.26 µg/mL respectively (Fig. 3). The positive control, ascorbic acid exhibited significant free radical scavenging activity with IC_50_ of 4.8 ± 0.12 µg/mL. It’s worth to note that any modification on the basic structure of erythraline (4) resulted in decrease antioxidant activity. Furthermore, a structural comparison of compounds **1**–**4** and their respective antioxidant activities revealed that the presence of a carbonyl group at C8 leads to a decrease in the antioxidant activity of Erythrina alkaloids. This observation aligns with previous research, which reported that erysotramidine and erytharbine, both possessing a carbonyl group at C8, exhibited lower antioxidant activity compared to erythrartine. Unlike the former compounds, erythrartine lacks a carbonyl group at C8 and instead features a hydroxyl group at C11, contributing to its higher antioxidant activity^14,17^.

Furthermore, we conducted DFT calculation to shed light on how the electronic properties of the isolated compounds corelate with their respective free radical scavenging activities. Global descriptive parameters pertain to parameters that offer insights into a compound’s reactivity and responses when subjected to changes in external conditions^32^. These parameters include ionization potential (I), electron affinity (A), hardness (η), softness (σ), electronegativity (χ), chemical potential (μ) and electrophilicity index (Ω)^33^. These parameters provide valuable knowledge about the electronic and structural properties of the studied compound^34^. In this study, the concept of energy-vertical method was considered to compute the parameters related to the radical scavenging activity of the isolated alkaloids both in gas and ethanol phase, and the corresponding values are provided in Table 2.

**Table 2:** Global descriptive parameter values of Compound **1**–**4.**

Electronic properties (eV)	Compounds			
	(1)	(2)	(3)	(4)
Gas phase				
Ionization potential (I)	7.2116	7.7258	7.2313	7.1690
Electron affinity (A)	0.6463	0.3074	− 0.250	− 0.3188
Hardness (η)	3.2827	3.7092	3.7406	3.7438
Softness (σ)	0.1523	4.0166	3.4907	3.4250
Electronegativity (χ)	3.9289	0.1348	0.1337	0.1335
Chemical potential (μ)	− 3.9299	− 4.016	− 3.491	− 3.4250
Electrophilicity index (Ω)	7.7184	8.0665	6.0924	5.8653
Electron donating power (ω^–^)	4.7262	4.6467	3.8416	3.7471
Electron accepting power (ω^+^)	0.7971	0.6301	0.3509	0.3221
Ethanol				
Ionization potential (I)	5.6812	6.0510	5.8319	5.4294
Electron affinity (A)	2.2985	2.0469	1.2792	1.2680
Hardness (η)	1.6913	2.0021	2.2764	2.0807
Softness (σ)	3.9898	4.0490	3.5556	3.3487
Electronegativity (χ)	0.2956	0.2497	0.2196	0.2403
Chemical potential (μ)	− 3.9898	− 4.0490	− 3.5556	− 3.3487
Electrophilicity index (Ω)	7.9594	8.1971	6.3210	5.6068
Electron donating power (ω^–^)	6.9124	6.3690	4.8391	4.6291
Electron accepting power (ω^+^)	2.9225	2.3201	1.2836	1.2804

All the isolated alkaloids exhibited a shared skeletal structure, prompting an intriguing exploration of their concomitant electronic and structural properties of these isolated alkaloids. The lower ionization potentials of erythrinine (**3**) and erythraline (**4**) suggest their greater ease of electron donation compared to cristamidine (**1**), and 8-oxoerythraline (**2**), which is critical for free radical scavenging^35^. Additionally, the lower electron affinity values of erythrinine (**3**) and erythraline (**4**) indicate a reduced tendency for these compounds to accept electrons^36^, reinforcing their role as efficient electron donors. The electron donating power and electron accepting power further support these findings. Erythrinine (**3**) and erythraline (**4**), with lower electron donating powers and electron accepting power, demonstrate enhanced free radical scavenging activities. Their lower electron donating powers suggest and lower electron accepting power suggest that both erythrinine (**3**) and erythraline (**4**) tend to act as electron donor, whereas cristamidine (**1**), and 8-oxoerythraline (**2**) tend to act as electron acceptor^32^. Moreover, the correlation of these electronic properties with the results of the DPPH radical scavenging assay supported the previous finding that compounds with a higher tendency to donate electrons have better DPPH radical scavenging activity than those with a higher tendency to accept electrons^37^.

## Conclusion

Our research aimed to identify the alkaloids present in the twigs of *E. crista-galli* and evaluate their free radical scavenging activities through both in vitro and in silico studies. We tentatively identified twelve alkaloids from the twigs, with four of these alkaloids namely crystamidine (**1**), 8-oxoerythraline (**2**), erythrinine (**3**), and erythraline (**4**) being isolated and structurally confirmed through NMR and MS spectroscopy. The four alkaloids exhibited varied free radical scavenging activities as determined by their IC_50_ values. Compound **3** and **4**, characterized by tertiary amine functional groups, demonstrated superior antioxidant activities (IC_50_ of 182.5 ± 5.3 µg/mL and 285 ± 10.9 µg/mL, respectively) compared to alkaloids **1** and **2**, which possess amide functional groups (IC_50_ of 681.4 ± 20.2 µg/mL and 868.2 ± 0.26 µg/mL, respectively). The global descriptive parameters suggest that compound **1** and **2** tend to act as electron acceptors, whereas compound **3** and **4** tend to act as electron donors. The correlation of these electronic properties with the results of the DPPH radical scavenging assay suggests that compounds with a higher tendency to donate electrons exhibited higher DPPH radical scavenging activity than those with a higher tendency to accept electrons.

## Supplementary Information

Supplementary Information.

## Data Availability

All data generated or analysed during this study are included in this published article.
