# Supplementary material for: Metabolite profiling and free radical scavenging activity studies of alkaloids from Erythrina crista-galli twigs through in vitro and in silico analysis
Source: Sci Rep. 2025 Oct 2;15:34376. doi: 10.1038/s41598-025-17130-x (PMC12491411; doi:10.1038/s41598-025-17130-x)
Supplement: Supplementary file 1 — Supplementary Information. [file 41598_2025_17130_MOESM1_ESM.docx]

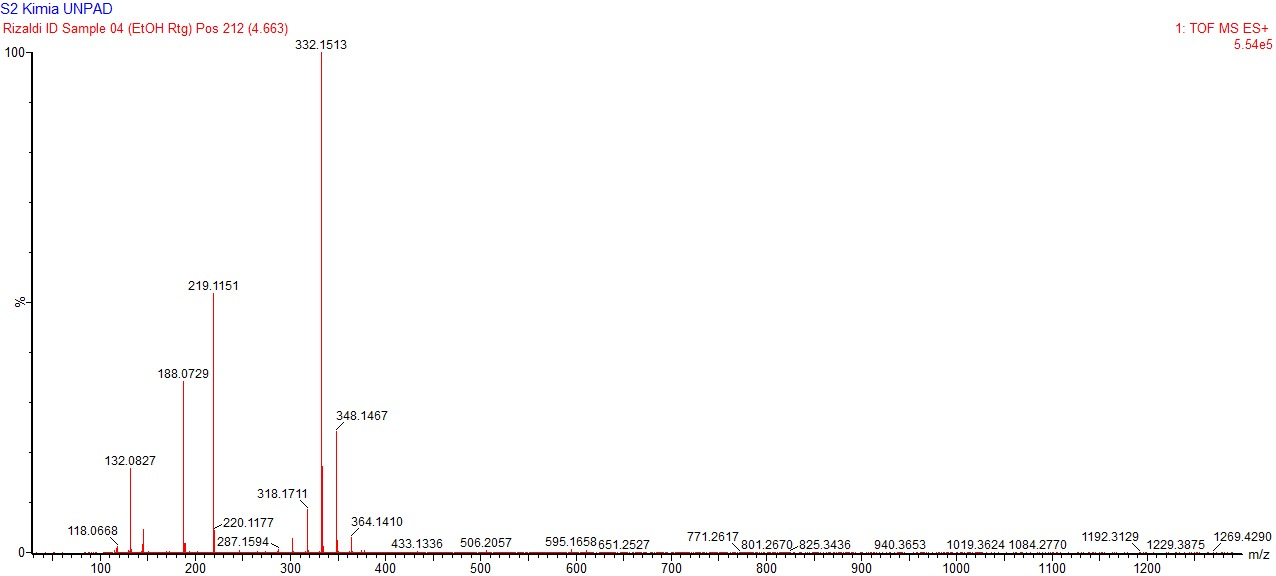


Figure S1 Mass spectrum in RT 4.66


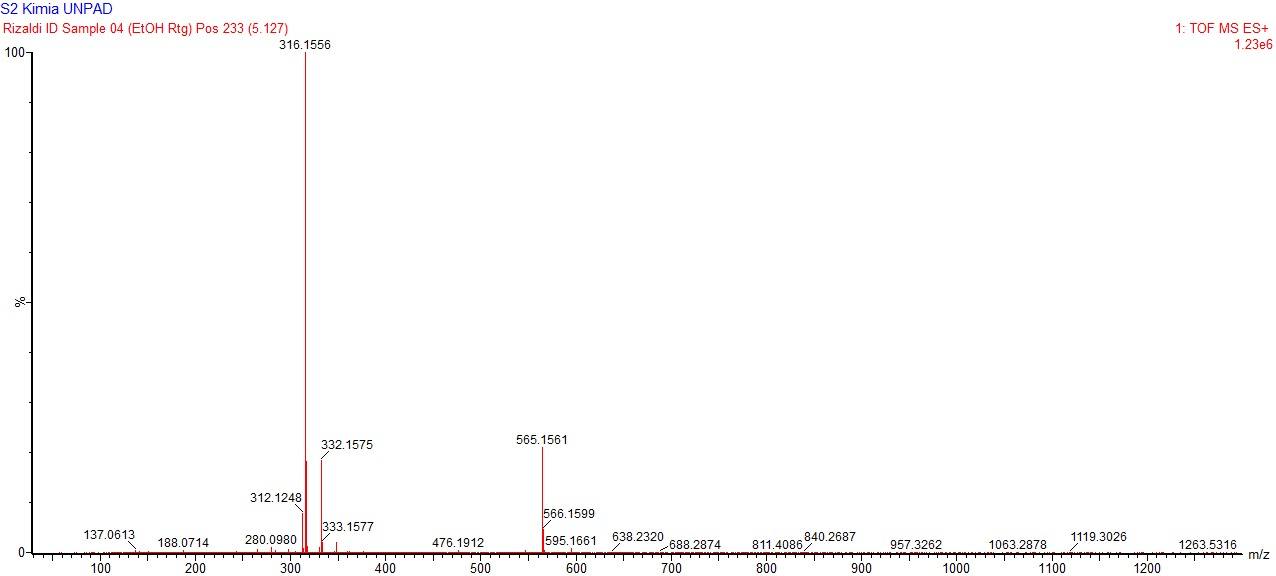


Figure S2 Mass spectrum in RT 5.13


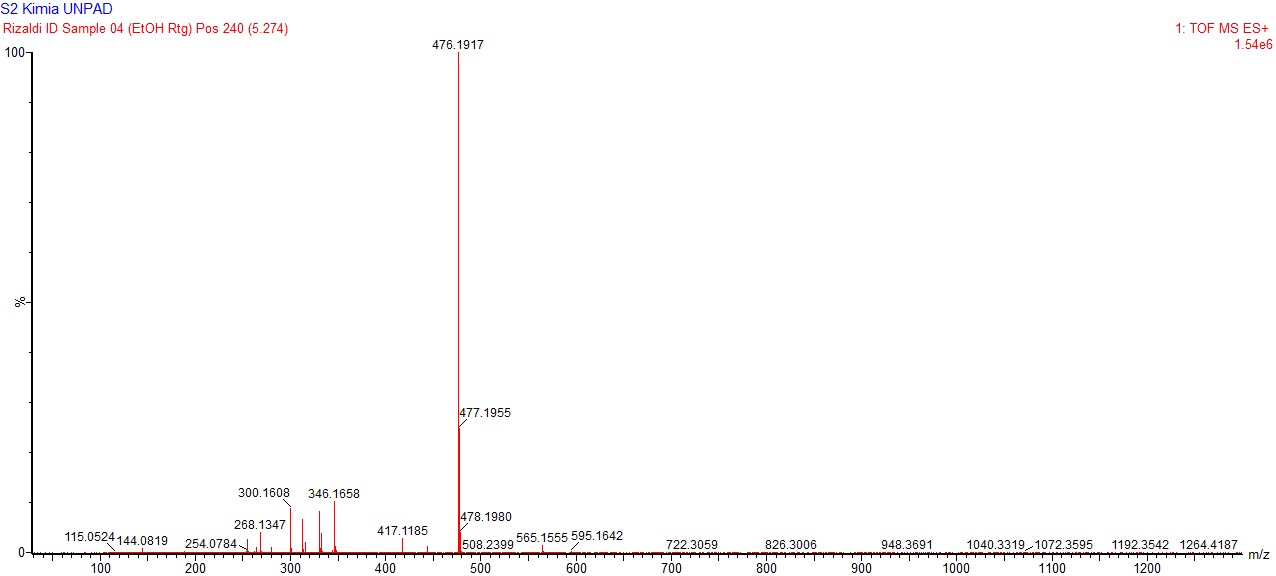


Figure S3 Mass spectrum in RT 5.27


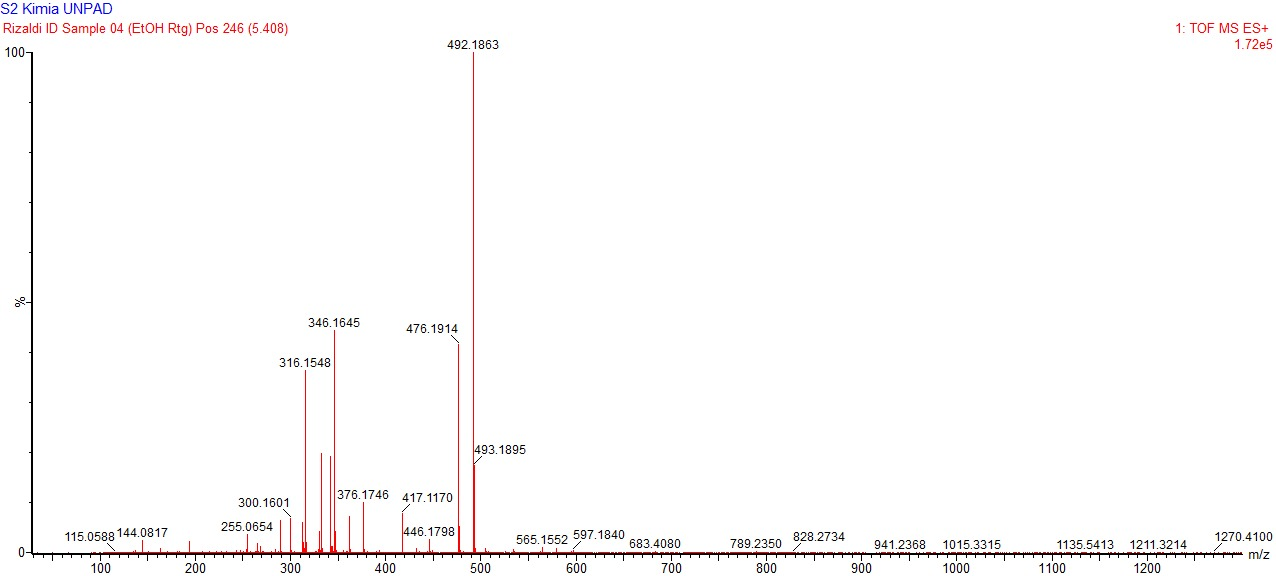


Figure S4 Mass spectrum in RT 5.41


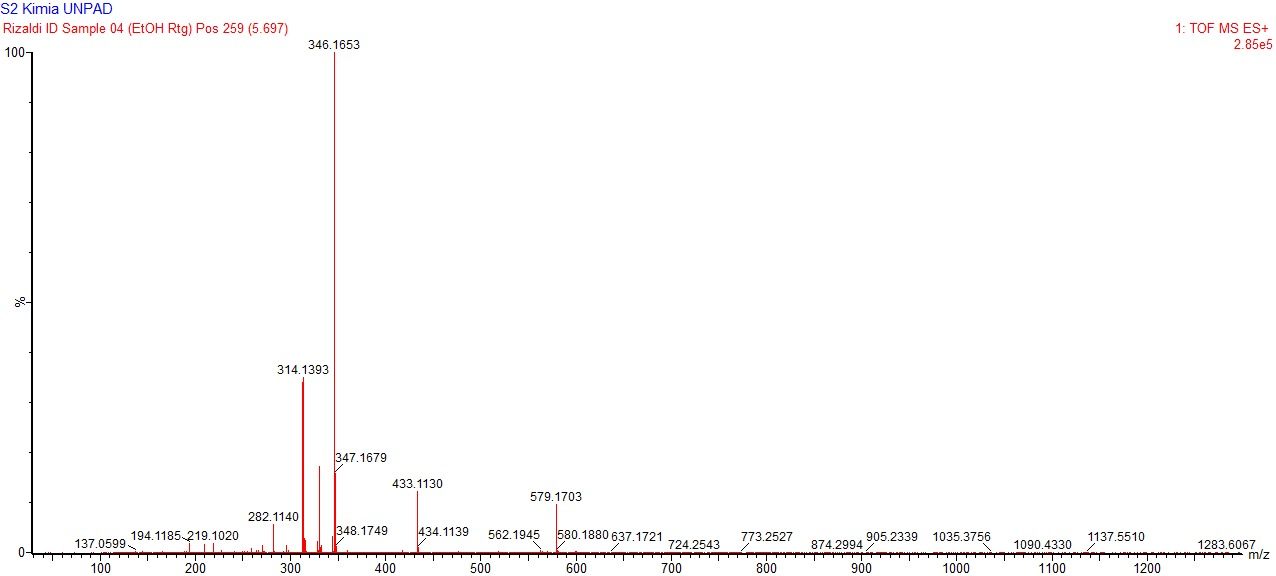


Figure S5 Mass spectrum in RT 5.69


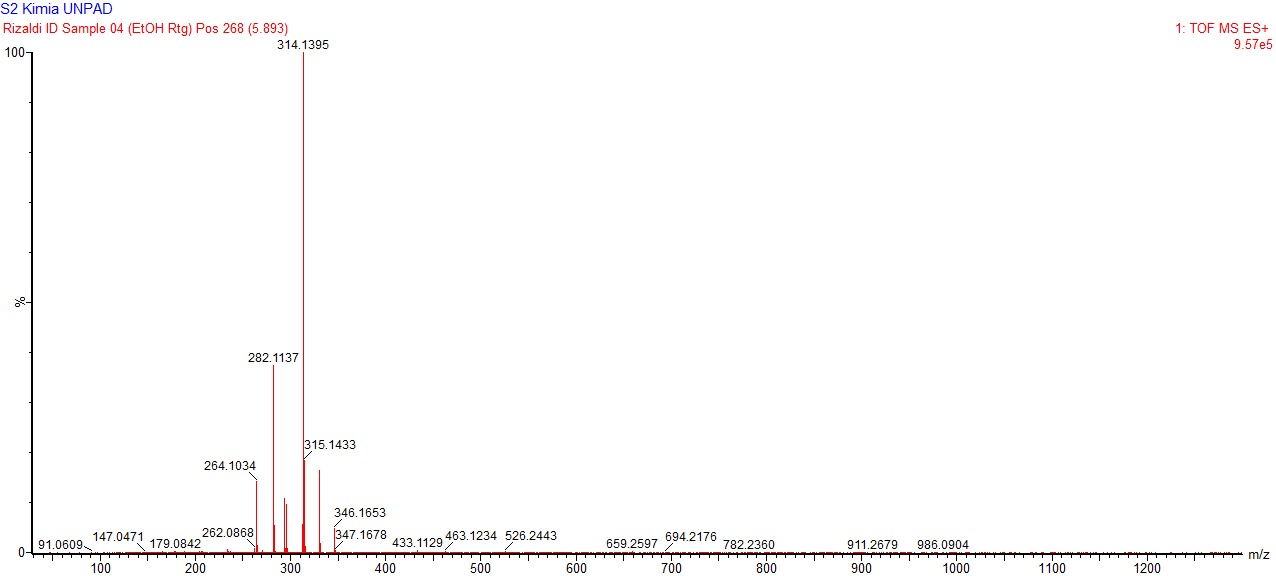


Figure S6 Mass spectrum in RT 5.89


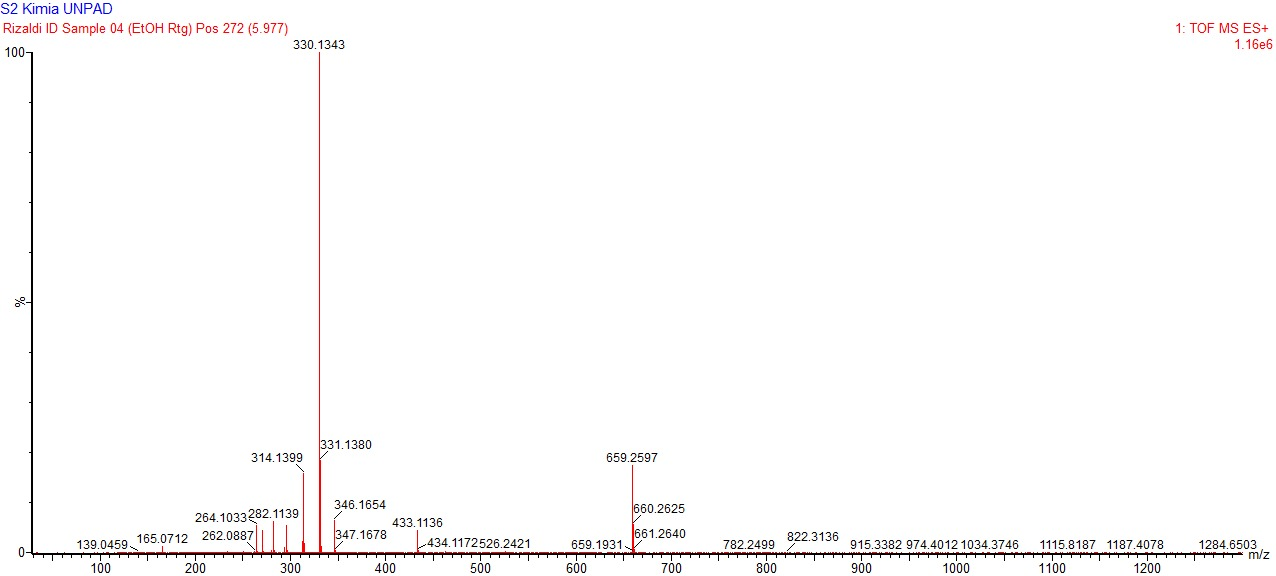


Figure S7 Mass spectrum in RT 5.97


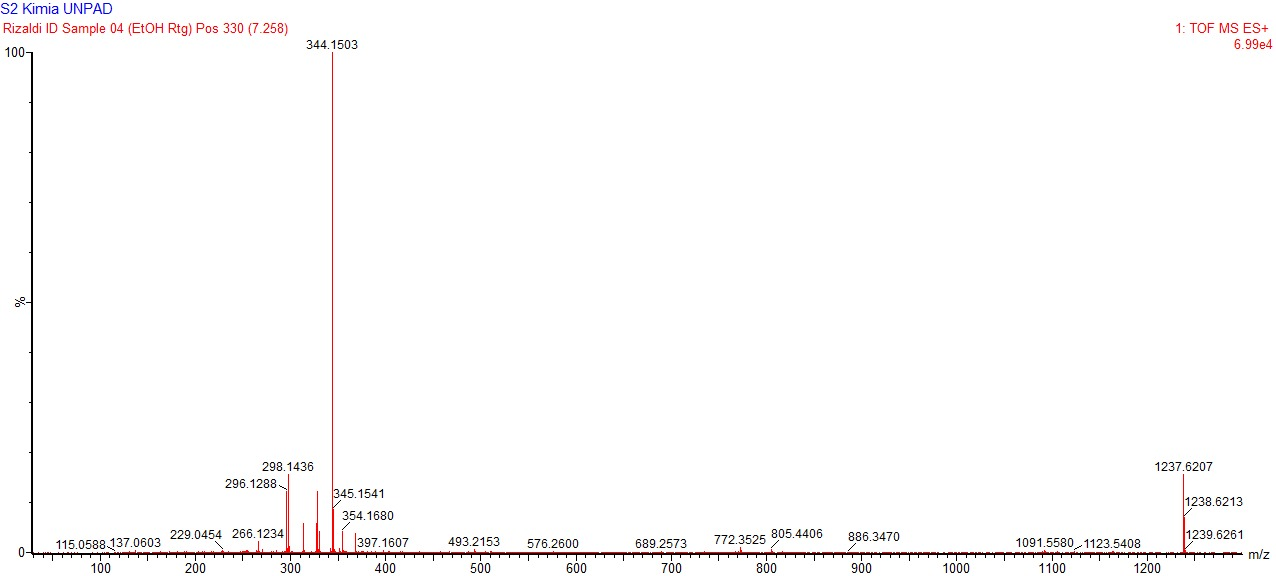


Figure S8 Mass spectrum in RT 7.26


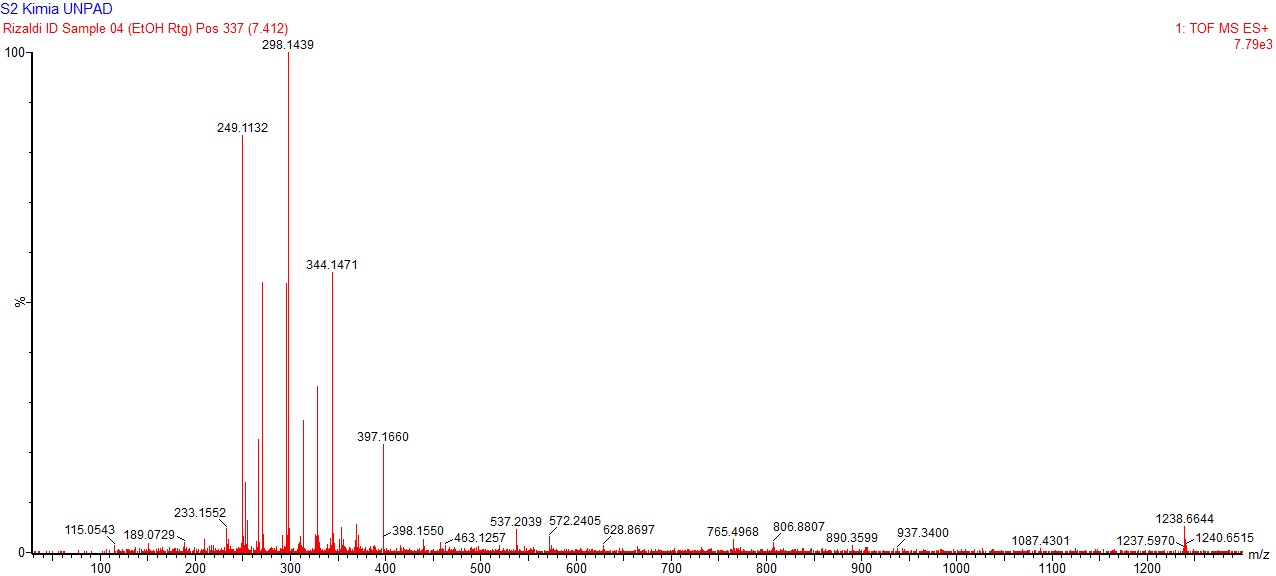


Figure S9 Mass spectrum in RT 7.41


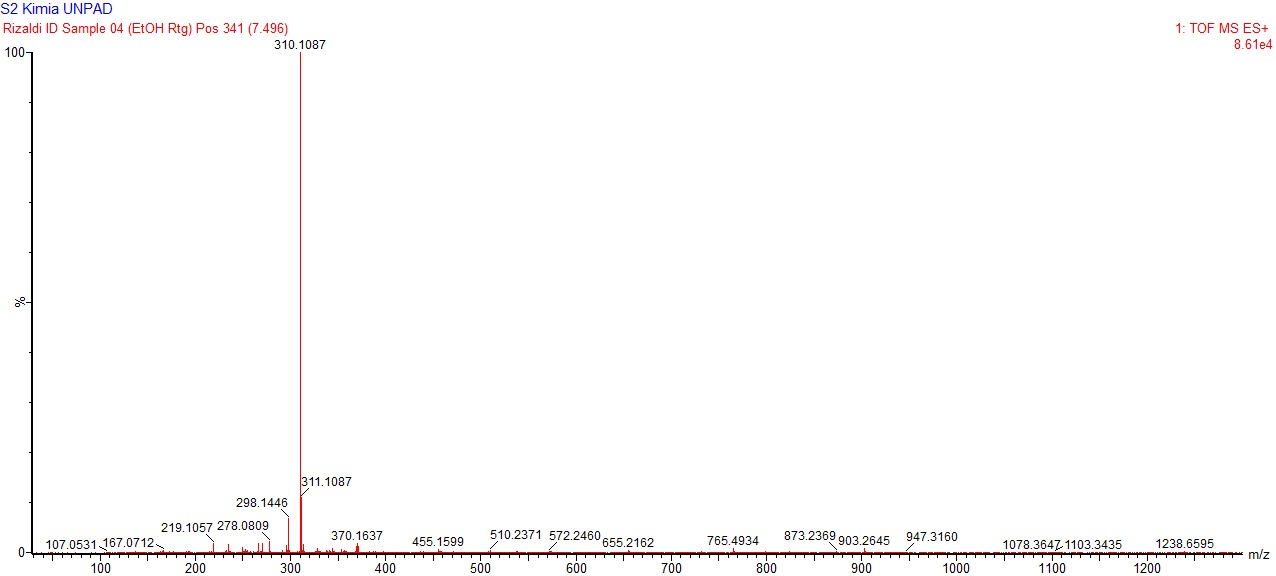


Figure S10 Mass spectrum in RT 7.49


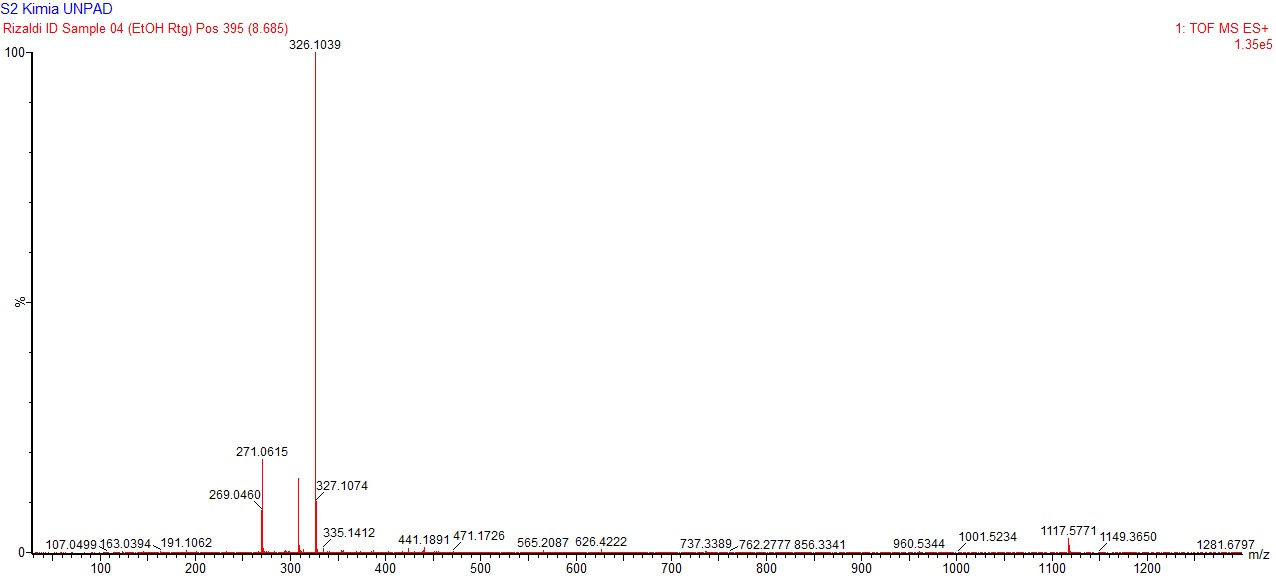


Figure S11 Mass spectrum in RT 8.61


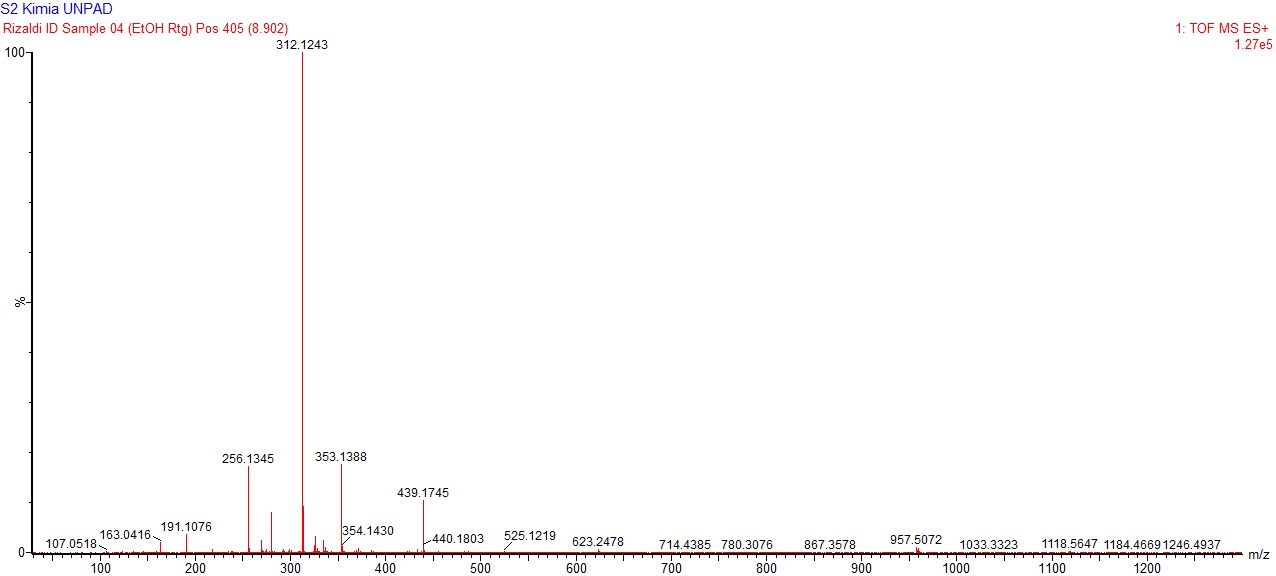


Figure S12 Mass spectrum in RT 8.90
